# Antenatal Sonographic Diagnosis of Pharyngeal Teratoma: Our Experience of a Rare Case with Review of the Literature

**DOI:** 10.1155/2009/180643

**Published:** 2009-04-02

**Authors:** M. Varras, Ch. Akrivis, Ch. Plis, G. Tsoukalos

**Affiliations:** ^1^Obstetrics and Gynecology Department, Tzaneio General State Hospital, Zanni and Afendouli, Piraeus, Greece; ^2^Platonos 33, Politia (Kifisia) 14563, Athens, Greece; ^3^Obstetrics and Gynecology Department, G. Chatzikosta General State Hospital, Makrygianni Avenue, Ioannina 450 01, Greece

## Abstract

*Background*. Teratomas are the most common tumors. They are usually localized in the sacrococcygeal area, while the pharyngeal localization is very rare. The number of cases of stomatopharyngeal teratomas detected prenatally via sonography is very small. *Case Report*. We present the case of a 24-year-old primipara at 18 weeks' gestation, that at the routine ultrasound scan, the fetus was found with an echogenic mass, filling the stomatopharyngeal cavity and protruding from the mouth. Other abnormalities were not found. Termination of pregnancy was achieved using misoprostol. A female stillborn fetus with a weight of 250 g and length of 25.5 cm was delivered. The postmortem and pathologic examination confirmed the diagnosis. *Conclusion*. Pharyngeal teratomas can be diagnosed with the use of ultrasounds in utero facilitating parents' counseling in early time.

## 1. Introduction

Teratomas are tumors developed from totipotent stem cells and contain tissues of ectodermal, endodermal, and mesodermal origin [1]. A fetal teratoma originating from the base of the skull is called pharyngeal teratoma or epignathus [2, 3]. It is a rare, congenital teratoma that is attached to an intraoral surface, most often palatal or pharyngeal and is not normally associated with other anomalies [3–6]. It usually carries a poor prognosis [7]. Complications include polyhydramnios, preeclampsia, preterm delivery, and respiratory compromise after birth due to upper airway obstruction by the mass [5, 7]. We report a case of pharyngeal teratoma with ultrasonographic and postmortem descriptions and review literature.

## 2. Case Report

**Figure 1:**
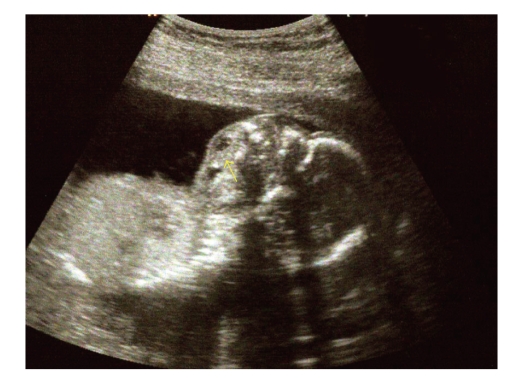
Longitudinal sonogram of the fetal head at 18 weeks' gestation shows a complex mass (arrow) filling the oropharynx and spilling out the fetal mouth.

**Figure 2:**
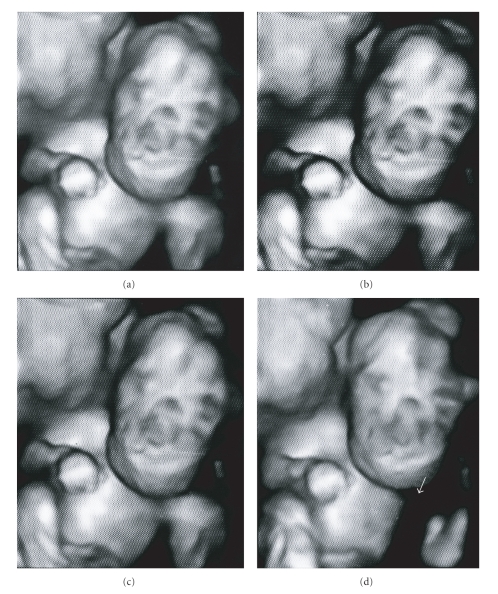
The 3D ultrasound scan shows the relationship of the mass with the mouth and the face.

**Figure 3:**
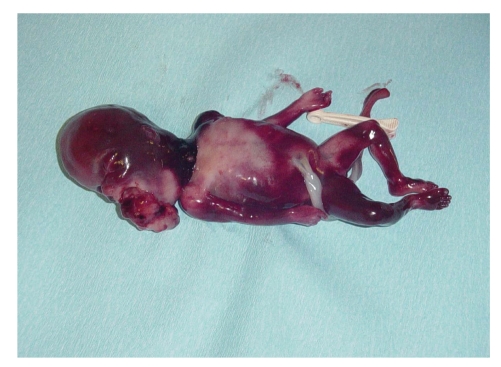
Photograph of the stillborn infant shows the tumor protruding through the open oral cavity.

**Figure 4:**
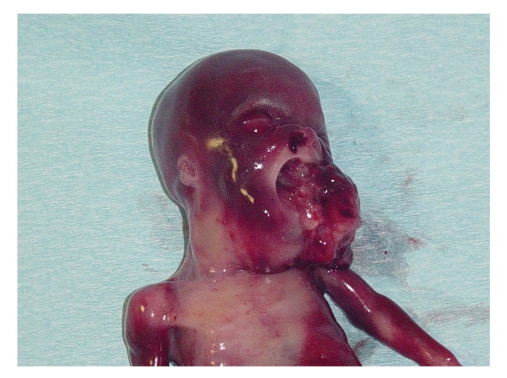
Photograph of the stillborn infant shows the tumor protruding through the open oral cavity.

A healthy 24-year-old primigravida was presented to the Department of Obstetrics and Gynaecology, Tzaneio General State Hospital, Piraeus, Greece, for the first visit of her pregnancy. The combined nuchal translucency and biochemical markers screening test for aneuploidies between the 11 and 13^+6^ weeks of pregnancy or the ultrasound fetal measurement and quadrant blood test (AFP, free beta-hCG, free oestriol, and indibin A) during the second trimester of pregnancy were not performed. She was healthy, used no medication, had not been exposed to known radiation and there were no malformed neonates within her family. Sonographic examination revealed a single live fetus, 17^+5^ weeks of gestation with biparietal diameter of 44 mm, head circumference of 155 mm, femur length of 26 mm, and abdominal circumference of 144.5 mm. There was a complex mass filling the oropharynx and emerging from the fetal mouth (Figures 1  and 2). The placenta was located on the uterine fundus. The amount of amniotic fluid was increased, but the rest of the examination was normal. The differential diagnosis included hemangioma, teratoma, or a tumor of neural origin. After appropriate counseling concerning the poor prognosis, the parents decided to terminate the pregnancy at the 18 weeks' gestation. Labor was induced with vaginal misoprostol and a 250 g stillborn female neonate was delivered with a tumor protruding through the open oral cavity (Figures 3  and 4).

The autopsy revealed an exophytic palatine mass that measured 6 × 4 × 2 cm and was attached to the oropharynx and nasopharynx. Histological analysis confirmed the diagnosis of pharyngeal teratoma. There were no additional malformations or fetal chromosomal aberrations. Also, parental chromosomes were normal. The parents were reassured that this was a sporadic occurrence and that recurrence risk was extremely low.

## 3. Discussion

Teratomas are the most common congenital neoplasms, which account for 25–35% of all neonatal tumors and present with an incidence of 1 in 20 000 to 1 in 40 000 live births [2, 8, 9]. However, teratomas involving the pharynx and the base of the skull present with a frequency of less than 1% of all congenital teratomas [3]. Teratomas usually occur more frequently in females than in males with a ratio of 3:1 [2]. They generally contain derivatives of two or three germ cell layers and may be classified as mature, immature or malignant based on their histopathologic appearance and behavior [9]. Brain tissue is the most frequent component, but cartilage, bronchial epithelium, and ependymalined cysts are common [9]. Teratomas involving the pharynx and the base of the skull arise from cells of the oral membrane, around the pouch of Rathke, and are in the vast majority benign [9, 10]. There is no evidence to suggest environmental risk factors in the pathogenesis of pharyngeal teratoma [2]. Furthermore, there are no karyotype abnormalities involved and these lesions are not thought to be inherited in a Mendelian or polygenic fashion [2, 7, 11].

The clinical presentation of congenital pharyngeal teratoma varies. Maternal serum *α*-fetoprotein assays are usually used to screen for neural tube defects and autosomal trisomies. However, elevated levels of maternal serum *α*-fetoprotein have also been reported in pregnancies with fetal defects such as tracheoesophageal fistula, esophageal obstruction, sacrococcygeal teratoma, and anorectal atresia. The physiological mechanism of maternal serum *α*-fetoprotein elevation in teratomas is probably the loss of fetal serum into the amniotic cavity combined with esophageal and tracheal obstruction by the mass [9]. The high levels of maternal serum *α*-fetoprotein can lead to a sonographic investigation and the diagnosis of the tumor [2]. It may also be detected on routine sonographic examination, as in our case. Teratomas generally appear as both solid and cystic on sonograms [2]. The solid component is often nonhomogenous, composed of tissues of different density such as cartilage and liver and may contain calcifications that may be tooth or bone. The cystic spaces are irregular and are formed by cavities lined with neural, respiratory, gastrointestinal or squamous epithelium [2]. These tumors frequently cause increase of the amniotic fluid as in our case, due to inability of the fetus to swallow the amniotic fluid. Also, these tumors frequently cause palatal clefting [10]. In our case cleft was not detected in the palate and this was also confirmed by the postmortem examination. In addition, in the present case, 3D ultrasound scan was helpful in assessing the external fetal anatomy and had provided images that were understandable by parents thus facilitating counseling (Figure 2).

The prognosis of pharyngeal teratoma is poor [2, 11]. It depends mainly on the size of the lesion and the involvement of vital structures [2]. In two reviews comprising 33 neonates with epignathus, 12 expired in utero or immediately after birth [12, 13]. Most deaths were attributed to airway obstruction. Also, difficulty in feeding is a common symptom for neonate with pharyngeal teratomas.

## 4. Conclusions

Pharyngeal teratomas can be suspected with the use of ultrasounds in utero facilitating parents' counseling in early time. However, the final diagnosis of pharyngeal teratomas is ultimately a pathological diagnosis. If the parents decide to continue the pregnancy, consideration should be given utilizing the exit procedure for air management at delivery. Finally, it is important to reassure parents that they are not at increased risk of bearing another child with these lesions.
